# A baboon model for endometriosis: implications for fertility

**DOI:** 10.1186/1477-7827-4-S1-S7

**Published:** 2006-10-09

**Authors:** Julie M Hastings, Asgerally T Fazleabas

**Affiliations:** 1 Department of Obstetrics and Gynecology (MC808), College of Medicine, University of Illinois at Chicago, 820 S Wood Street, Chicago, Illinois, 60612, USA

## Abstract

Endometriosis is one of the most common causes of chronic pelvic pain and infertility in women in the reproductive age group. Although the existence of this disease has been known for over 100 years our current knowledge of its pathogenesis and the pathophysiology of its related infertility remains unclear. Several reasons contribute to our lack of knowledge, the most critical being the difficulty in carrying out objective long-term studies in women. Thus, we and others have developed a model of this disease in the non-human primate, the baboon (*Papio anubis*). Intraperitoneal inoculation of autologous menstrual endometrium results in the development of endometriotic lesions with gross morphological characteristics similar to those seen in the human. Multiple factors have been implicated in endometriosis-associated infertility. We have described aberrant levels of factors involved in multiple pathways important in the establishment of pregnancy, in the endometrium of baboons induced with endometriosis. Specifically, we have observed dysregulation of proteins involved in invasion, angiogenesis, methylation, cell growth, immunomodulation, and steroid hormone action. These data suggest that, in an induced model of endometriosis in the baboon, an increased angiogenic capacity, decreased apoptotic potential, progesterone resistance, estrogen hyper-responsiveness, and an inability to respond appropriately to embryonic signals contribute to the reduced fecundity associated with this disease.

## Introduction

Endometriosis, the presence of endometrial glands and stroma outside of the uterine cavity, is one of the most common causes of chronic pelvic pain and infertility: it affects 1 in 10 women in the reproductive-age group [1]. This incidence increases up to 30% in patients with infertility [2]. Several theories have been proposed to explain the etiology of endometriosis; however, the most widely accepted hypothesis for the development of endometriosis is Sampson's theory of retrograde menstruation, in which fragments of menstrual endometrium are refluxed through the fallopian tubes into the peritoneal cavity [3]. However, although retrograde menstruation occurs in 70–90% of women in the reproductive age group, endometriosis is only diagnosed in 10% of this population [1,4]. While the existence of this disease has been known for more than one hundred years, our current knowledge of the pathogenesis of this spontaneous evolution and the pathophysiology of the related infertility remains unclear. Several factors contribute to this lack of understanding. Most significantly, at the time of clinical presentation, most women have established disease; furthermore, the subsequent time taken to diagnosis can be extremely long [5,6]. Individual differences in progression and symptomatology of the disease create difficulties in conducting objective long-term studies in women. Finally, endometriosis only occurs naturally in humans and non-human primates. Thus, because ethical and practical considerations limit studies in the human, an appropriate animal model needs to be developed.

Experimental models of endometriosis have been developed in immunocompromized rodents that allow the transplantation of human endometrial tissue with limited graft rejection [7-9]. Other investigators perform allotransplantation of uterine endometrium from syngeneic mice [10]. These models are advantageous because they have limited cost and large study groups can be investigated. Moreover, these animals are usually "inbred" and experimental results are more reproducible than those seen in "out-bred" human and non-human primate subjects, with less biological variation. However, while rodents provide an excellent first-line approach to investigate the etiology of this enigmatic disease, there are several limitations of these models, least of which are the lack of a menstrual cycle and the spontaneous development of endometriosis.

Spontaneous endometriosis has been reported in the rhesus macaque (*Macaca mulatta*), the Japanese macaque (*Macaca fuscata*), the pig-tailed macaque (*Macaca nemestrima*), and the Kenya baboon (*Papio doguera*), leading researchers to evaluate the use of the non-human primate as a model to investigate endometriosis [11-14]. It was proposed that iatrogenically induced retrograde menstruation would result in the development of endometriosis, supporting the hypothesis of Sampson. Indeed, endometriosis was experimentally induced in rhesus macaques by surgical diversion of the cervix into the abdomen; however, endometriosis was found in only 50% of the animals [15,16]. Supracervical ligation impeded uterine outflow in baboons, illustrated by a decreased duration of antegrade menstruation and increased retrograde menstruation. Although this study size was small, histologically-confirmed endometriosis was observed in the two animals as early as three months after supracervial ligation [17]. Increased levels of endometriosis were observed in rhesus macaques subjected to prolonged exposure to the dioxin 2,3,7,8-Tetrachlorodibenzo-*p*-dioxin (TCCD); however, the follow-up time of this study was ten years [18].

More recent pioneering work from D'Hooghe and co-workers demonstrated that endometriosis develops naturally in a subset of female olive and yellow baboons (*Papio anubis *and *Papio cynocephalus*, respectively) maintained in captivity and that the incidence of clinical and biopsy-proven endometriosis in the baboon is a progressive disease that increases with the duration of captivity [19,20]. D'Hooghe *et al*., further demonstrated that experimental induction of endometriosis by inoculation of endometrial currettings into the peritoneal cavity produced both readily recognizable pelvic endometriotic lesions that were macroscopically similar to those seen in women with spontaneous endometriosis and an increased rate of infertility [21,22].

We now report studies using a modified version of this baboon model of induced endometriosis, to demonstrate the aberrant expression of several genes in the eutopic and ectopic endometria of these animals. We propose that such changes may mediate the establishment and progression of endometriosis and the reduced fecundity associated with this enigmatic disease.

## An Experimental Model of Endometriosis in the Baboon

Endometriosis was experimentally induced in female *Papio anubis *baboons, with documented regular menstrual cycles, by intraperitoneal inoculation with menstrual endometrium on two consecutive menstrual cycles. Menstrual endometrium (0.84 ± 0.22 g) was harvested on day 1 or 2 of menses using a Unimar Pipelle just prior to laparoscopy. The peritoneal cavity and reproductive organs were visualized by laparoscopy and the absence of any lesions or adhesions was documented by video recording. Under laparoscopic guidance, menstrual tissue was deposited from the Pipelle at four sites; the pouch of Douglas, the uterine fundus, the cul de sac, and the ovaries. At the subsequent mense, the animals underwent a second laparoscopy and endometrial reseeding at the same ectopic sites. The progression of disease was monitored in animals by several consecutive laparoscopies over a period of 15 months after inoculation, during the window of uterine receptivity (days 9–11 post-ovulation (PO) in the baboon). Following each laparoscopy a laparotomy was performed when ectopic endometriotic lesions and matched eutopic endometrium was harvested by endometriectomy.

**Figure 1:**
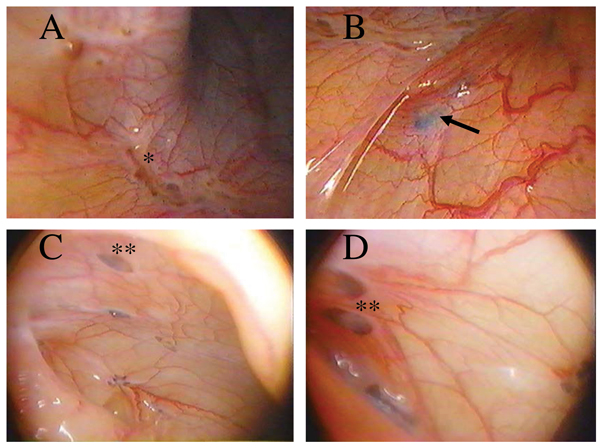
Laparoscopic Evaluation of Lesions in a Baboon Model of Experimental Endometriosis. Visualization of the peritoneal cavity by laparoscope demonstrated the presence of powder burns (identified by a single asterisk) and blue (identified by the arrow) and chocolate lesions (identified by the double asterisks) within three (A,B) and six (C,D) months of induction of disease.

**Figure 2:**
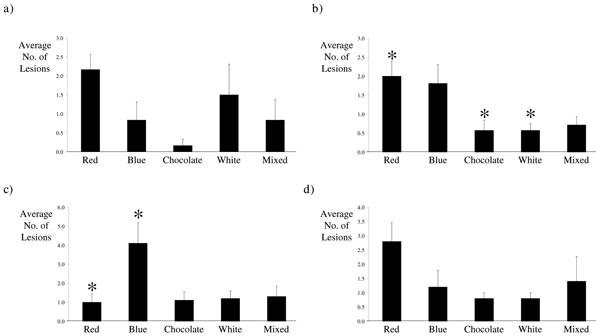
Characterization of Lesions Visualized by Laparoscope in Baboons Experimentally Induced with Endometriosis. Laparoscopic evaluation of the peritoneal cavity of baboons a) one month following induction of disease revealed similar levels of red, blue, chocolate, white, and mixed lesions. The peritoneal cavity contained a significantly higher number of red lesions b) three months following induction of endometriosis; however, c) 6 months after inoculation with menstrual endometrium significantly higher numbers of blue lesions were present in the peritoneal cavity of baboons with induced disease. Similar numbers of each lesions type were visible by laparoscopy d) 15 months after induction of endometriosis. Columns represent the mean number of red, blue, chocolate, white, and mixed lesions visualized by laparoscope a) 1 month (n = 6), b) 3 months (n = 21), c) 6 months (n = 10), and d) 15 months (n = 5) after induction of disease. Error bars represent the standard error of the mean. (*, p < 0.05, Kruskal-Wallis ANOVA, with Dunn's Correction for multiple testing.)

**Figure 3:**
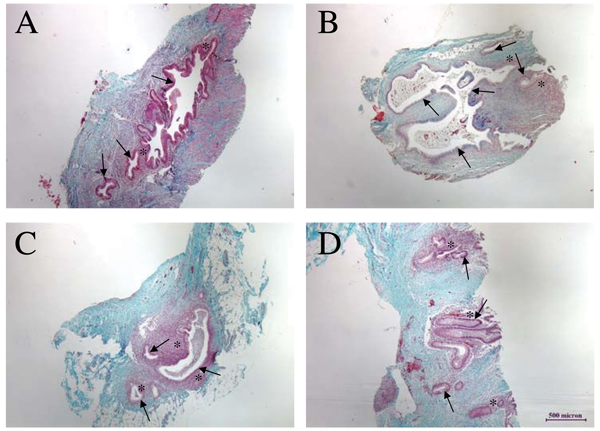
Histological Evaluation of Endometriotic Lesions. Panel A is a section through a chocolate lesion harvested from the uterine surface at three months. Panels B and C are sections through blue lesions obtained at six months of disease from the uterine fundus (B) and 12 months of disease in peritoneal fat (C). Panel D is a section through a red lesion obtained at 16 months of disease from the peritoneal wall. The presence of endometriotic tissue is histologically confirmed by the presence of endometrial glands (indicated with arrows) and stroma (highlighted with asterisks): the stroma surrounding glands of endometriotic lesions ranges from only a few cells (as in B) to several cells in depth (as in C).

Intraperitoneal inoculation resulted in the formation of endometriotic lesions with gross morphological characteristics similar to those seen in women (Figure 1). The development of these endometriotic lesions was observed in all 24 animals that have undergone intraperitoneal inoculation. The number and type of lesions ranged between animals, but on average we observed two red, three blue, one chocolate, one white and two lesions of mixed pigmentation during each laparoscopy. Significantly more red lesions, which are thought to represent the most active site of disease, were observed three months following inoculation with menstrual endometrium (Figure 2b), while at six months of disease significantly more blue endometriotic lesions were present (Figure 2c). Thereafter similar levels of red, blue, chocolate, white, and mixed lesions were observed, although there was a trend towards an increased number of red lesions, indicating that the disease is still active (Figure 2d). Morphologically, 67% of the ectopic lesions harvested at the time of laparotomy contained both endometrial glands and stroma (Figure 3).

The effect of endometriotic lesions on markers of uterine receptivity was evaluated both in cycling baboons between days 9–11 post-ovulation (PO) and those treated with chorionic gonadotrophin (CG) to simulate pregnancy [23,24]. All laparoscopies and laparotomies were performed during the window of uterine receptivity (days 9–11 PO) in the baboon). Control endometria were similarly harvested from animals with no disease at days 9–11 PO or during the late proliferative phase of the cycle. In the following we first discuss developmental aspects of the endometriotic lesions, including their angiogenic potential, and then explore the impact this disease may have on receptivity and fertility.

## Development of endometriotic lesions is an invasive event

The first steps in the pathogenesis of endometriosis require attachment and invasion of the peritoneal lining by endometrial fragments. The mechanism by which invasion of the peritoneum occurs is not fully understood. Some investigators propose that the peritoneal mesothelium acts as a barrier to refluxed endometrium suggesting that endometrial attachment only occurs at sites of peritoneal damage [25]. However, other investigators have shown rapid attachment and invasion through peritoneal surfaces by endometrial stromal and epithelial cells. Subsequently, mesothelial cells are thought to integrate into the endometriotic tissues within the peritoneal layer by a process referred to as re-epitheliazation [26-29]. These processes mediating the initial establishment of disease are clearly invasive events that require breakdown of the peritoneal basement membrane and underlying extracellular matrix (ECM). Matrix metalloproteinases (MMPs) are essential for remodeling of the ECM in development, growth and repair of normal tissues and in inflammatory and degenerative diseases. Endometrial expression of the MMPs, and their tissue inhibitors (TIMPs), is normally tightly regulated throughout the menstrual cycle.

The precise role of the MMPs in the pathophysiology of endometriosis is not fully understood. However, we and others have reported aberrant patterns of MMP and TIMP protein and mRNA expression in eutopic and ectopic endometriotic tissues. Specifically, we have demonstrated high levels of MMP-7 in peritoneal endometriotic lesions in baboons with experimental endometriosis [30]. Furthermore, increased levels of both MMP-3 and MMP-7 were found in the eutopic endometrium of baboons with disease during the window of uterine receptivity when compared to cycle matched endometrium from control, disease-free animals [30]. These data were independently validated by Bruner-Tran *et al*., [31] who demonstrated increased MMP-3 and -7 protein and mRNA levels in the secretory eutopic endometrium of women with endometriosis. Other investigators have also shown increased levels of various MMPs and decreased levels of TIMPs in endometrium from patients with endometriosis compared to tissues from patients with no evidence of disease [32-37].

Blockade of MMP activity with TIMP-1 reduced the development of lesions in the experimental model of endometriosis in the ovariectomised nude mouse; furthermore, specific inhibition of "classical" MMPs (MMP-1, -2, -3, -7, and -13) with MMP inhibitor III reduced the development of endometriosis-like lesions in an *in vitro *invasion assay, the chicken chorioallantoic membrane (CAM) assay [37,38]. It has recently been proposed that endometrial MMP expression is regulated by tumor necrosis factor-α (TNFα) [33,39]. Studies in the rat and baboon have targeted TNFα activity for the prevention and treatment of endometriosis. Indeed, recombinant human TNF binding protein 1 (rhTBP1), the soluble form of TNFα receptor-type 1, reduced the size of endometriotic-like peritoneal lesions in the rat model of experimental endometriosis [40]. Furthermore, studies in the non-human primate demonstrated that antagonism of TNFα with either rhTBP1 or a fusion protein of TNF receptor-2 conjugated to human Fc antibody fragments (known as Etanercept) can reduce the development of experimental endometriosis or progression of spontaneous disease in baboons [41,42].

Therefore, we propose that the increased levels of MMPs present in the eutopic endometrium of women and baboons with endometriosis promote the invasiveness of endometrial fragments upon retrograde menstruation into the peritoneal cavity. Inhibition of development of lesions in both the *in vivo *rodent and baboon models of endometriosis and *in vitro *invasion assays by inhibition of MMPs further promotes the role of the MMPs in this disease. However, it is unclear whether the increased levels of these proteins are a cause or effect phenomenon of endometriosis.

## Angiogenic Profile of Endometriotic Tissues

Angiogenesis is the formation of new blood vessels from existing vessels and the process is tightly regulated in the adult. However, the primate endometrium undergoes profound vascular remodeling during each menstrual cycle and it has been proposed that angiogenesis is important in the pathophysiology of endometriosis. Indeed, endometriotic lesions appear to be surrounded by peritoneal blood vessels (Figure 1) and increased levels of numerous angiogenic factors are well documented in the peritoneal fluid of women with endometriosis [43-50]. The most potent angiogenic factor identified to date is vascular endothelial cell growth factor-A (VEGF). VEGF is thought to play a critical role in the vascular remodeling that occurs during each menstrual cycle in normal endometrium. Several investigators have demonstrated increased levels of VEGF in ectopic endometriotic lesions [51-55]. We have confirmed these findings in the baboon model of endometriosis. Whilst we demonstrated increased levels of VEGF in all ectopic lesions compared to control and eutopic matched endometriotic endometrium throughout progression of disease, maximal levels were observed in the more active red lesions [56].

More recently, the angiogenic factor CYR61, a member of the CCN family of growth factors involved in development, proliferation and tumorigenesis, has been shown to be up-regulated in eutopic and ectopic endometria of women with endometriosis [57]. Expression of CYR61 mRNA is rapidly induced in an immediate early response manner by a spectrum of stimuli including growth factors, cytokines and estrogens [58-60]. We have demonstrated cyclical regulation of CYR61 in baboon endometrium, with maximal mRNA levels observed in shed menstrual endometrium [56]. CYR61 protein, which is primarily localized in the epithelial and vascular compartments of the baboon endometrium, showed cyclical regulation in both its level and distribution. Specifically, CYR61 protein is diffusely distributed in glands from the proliferative phase but is located at the apical edge of endometrial glands from the secretory phase. Furthermore, CYR61 was immunolocalized to the endothelial cells of small arterioles throughout the cycle, while larger vessels and spiral arteries were only immunopositive for CYR61 in menstrual endometrium. CYR61 mRNA and protein levels were increased in a time dependent manner in the eutopic endometrium, during the window of receptivity, of baboons experimentally induced with endometriosis [56]. In eutopic endometria of diseased animals, maximal up-regulation of CYR61 was observed at three months following inoculation, with a mean 6.3-fold increase in mRNA levels compared to cycle matched control endometria. CYR61 mRNA and protein levels then decreased in eutopic endometria throughout progression of disease, with a mean 3-fold up-regulation 15 months after induction of disease. In the ectopic endometriotic lesions, mean expression levels of *CYR61 *mRNA were 3-fold greater than in eutopic endometrium of endometriotic animals and 11.7-fold greater than in eutopic endometrium from normal control animals [56]. The highest levels of CYR61 mRNA was observed in the highly vascularized red lesions, with a mean expression level of 18.46 (0.96 – 41.26 n = 8). White ectopic lesions, which are thought to represent the less active sites of disease, demonstrated the lowest levels of CYR61, with a mean expression of 0.74 (0.46, 1.01, n = 2). Immunohistochemical analysis of the ectopic lesions showed a similar distribution of CYR61 protein to that in eutopic endometrium, with immunostaining primarily in the glandular epithelial and vascular endothelial cells. The increased levels of CYR61 correlate with increased levels of VEGF in both the ectopic and eutopic endometrium of baboons with experimental endometriosis.

Increased levels of angiogenic factors such as CYR61 and VEGF in ectopic endometriotic lesions presumably promote an increase in the peritoneal vascular network, thereby facilitating implantation and viability of the refluxed endometrium. Indeed, the use of anti-angiogenic compounds, including soluble flt-1 (VEGF-receptor 1), anti-VEGF antibody, TNP-40, endostatin, and angiostatin have demonstrated the importance of angiogenic processes in the establishment of endometriosis in the mouse [61-67]. The impact of increased angiogenic activity within the eutopic endometrium is unclear. Increased levels of angiogenesis are well documented in the eutopic endometrium of women with endometriosis [51,52,68-70]. In addition to CYR61 and VEGF, we have demonstrated increased levels of the chemokine, eotaxin, which has been shown to stimulate angiogenic activity in human, mouse, rat, and chick endothelial cells, in the eutopic endometrium of baboons experimentally induced with endometriosis [71,72]. Jones *et *al., [73] demonstrated increased levels of eotaxin in women treated *in vivo *with the progestin-only contraceptive pill, which is associated with breakthrough bleeding. Increased levels of CYR61, VEGF, and eotaxin in eutopic endometriotic endometrium may increase the angiogenic potential of the tissue and further potentiate the development of lesions. Furthermore, an aberrant angiogenic profile may be detrimental to an implanting embryo, such that altered angiogenesis may, in part, mediate the infertility that is associated with endometriosis.

## Coordinated Gene Dysregulation in Eutopic Endometrium Leads to Reduced Fertility in Endometriosis

Multiple factors have been implicated in endometriosis-associated infertility, including distortion of the pelvic anatomy, abnormalities of hormone secretion and action, alterations in peritoneal fluid, and disorders of fertilization and immunoregulatory function [74-77]. Examination of eutopic endometrium from women with endometriosis has revealed defects, including dysynchrony of angiogenic phenotype as described above, ultrastructural abnormalities [78], and alterations in molecular markers of endometrial receptivity. The latter include αvβ3 integrin distribution patterns, steroid hormone receptors (SHRs), and HOXA10 gene expression, all of which are aberrant in the endometrium of women with endometriosis [68,79-88].

### Dysregulation of Steroid Hormone Receptors in Endometriosis

We have shown altered SHR protein distribution during the window of receptivity in baboons with endometriosis. Specifically, we have observed a time dependent reduction in estrogen receptor-α (ESR1) immunostaining within endometrial stromal cells from one month post induction of disease; this decrease reached statistical significance within six months of disease and remained throughout the time course of disease to 15 months. Estrogen receptor-β (ESR2) staining was reduced in both epithelial and stromal cells. Whilst we observed comparable levels of progesterone receptor-B (Pgr-B) in endometriotic and control eutopic endometria, diminished Pgr-A immunostaining of glandular epithelial cells was demonstrated in endometriotic tissues when compared to control tissues [89]. Unlike alterations in VEGF and CYR61, this aberrant SHR profile was not observed until six months following induction of disease. We propose that the diminished levels of ESR1, ESR2, and Pgr-A act in concert to create a uterine environment that is non-receptive to an implanting embryo. Estrogenic and progestogenic events are coordinated by complex paracrine interactions between endometrial stromal and epithelial cells [90]. We propose that reduced levels of ESR1 within endometrial stromal cells mediates the reduction of the estrogen-regulated gene Pgr-A in the endometrial epithelial cells. Many estrogen-regulated genes are thought to be suppressed in the secretory endometrium under the influence of progesterone. We propose that in the absence of Pgr-A within the epithelial compartment, estrogen-regulated genes, such as MMPs, VEGF, and CYR61, are no longer subjected to progesterone-mediated suppression, creating a uterine environment that is not conducive to the establishment of pregnancy.

### Aberrant Endometrial Immunological Responses in Endometriosis

Infertility has been associated with aberrant expression of immune modulators, including leukemia inhibitory factor (LIF), soluble gp130, and IL-11 [91-93]. Many studies have shown aberrant levels of cytokines in the peritoneal cavity of women with endometriosis compared to those of control women, including IL-1, IL-6, IL-10, p40, tumor necrosis factor-α (TNFα), and transforming growth factor-β (TNFβ) [49,94-97]. The chemotactic activity of peritoneal fluid of patients with endometriosis is higher than that of women with no disease [98]. Correspondingly, it is well documented that there is a greater number of macrophages in the peritoneal cavity of women with endometriosis than in that of women with no disease [99-101]. We proposed that the inflammatory peritoneal environment created by the presence of endometriotic lesions induces a uterine immunological environment that is not conducive to the establishment of pregnancy. Indeed, microarray analysis of human endometrium obtained during the window of receptivity has shown a 50-fold decrease in the mRNA levels of the immunosuppressive molecule Glycodelin A (GdA) in women with endometriosis compared to those without [102]. Furthermore, in eutopic endometrium CG failed to induce GdA in an *in vivo *simulated model of pregnancy in baboons with endometriosis [103]. GdA has multiple immunosuppressive effects and its abundant presence at the fetal-maternal interface suggests a role in protecting the embryo from maternal immune rejection [104-110].

To determine if the development of endometriosis is associated with an inflammatory uterine environment we examined the expression of a panel of immunological factors involved in the T helper (Th) 1, Th2, and Th3 pathways in the eutopic endometrium of baboons with and without endometriosis. Six months following induction of disease 11 of the 90 genes represented on the array were differentially expressed during the window of receptivity between control and endometriotic eutopic endometria: one gene was down-regulated and 10 were up-regulated. IL-1R1 mRNA levels were down-regulated 5-fold in eutopic endometriotic endometrium, while those of NFkB, eotaxin, and members of the activating protein-1 (AP-1) family, *junD*, c-*fos *and JNK2, were up-regulated 3.0-, 7.9-, 2.3-, and 1.8-fold, respectively [71]. Surprisingly, we saw no difference in the expression of factors thought to be important in inflammatory processes; this is most likely a result of the absence of a pre-decidual response in the baboon. Unlike the human, where a pre-decidual response in the secretory phase of the menstrual cycle is accompanied by an increased inflammatory cell infiltrate and stromal cell differentiation, the baboon requires the presence of the embryonic signal to undergo such changes.

As described above, IL-1R1 was down-regulated in the eutopic endometrium of baboons experimentally induced with endometriosis [71]. Studies in mice indicate that intraperitoneal injection of IL-1 receptor antagonist (IL-1Ra) prevents implantation by perturbations in the epithelial cell integrin expression [111]. Furthermore, cytokine-induced apoptosis of epithelial cells is blocked by the IL-1Ra [112]. Reduction of IL-1R1 in the eutopic endometrium of baboons with induced endometriosis may increase the proliferative index of the endometrium; furthermore, aberration of αvβ3 expression seen in women with endometriosis may be mediated by the reduction of IL-1β signaling in the absence of IL-1R1. We have previously shown the importance of IL-1β in the simulated model of pregnancy in the baboon, where antagonism of IL-1β with the IL-1Ra suppresses the effects of CG on the morphological transformation of the endometrium, such as the formation of the epithelial plaque and stromal cell differentiation into a decidual phenotype [113]. Therefore, we propose that the reduced levels of endometrial IL-1R1 observed in baboons with endometriosis provide further evidence that this endometrium is incapable of responding to and/or mediating signals generated from the implanting embryo.

NFκB mRNA levels were 3-fold greater in the eutopic endometrium of baboons with experimental endometriosis than in that of baboons with no disease [71]. NFκB is a ubiquitously expressed transcription factor that can be activated in a wide variety of cell types and has been shown to regulate the expression of a wide variety of genes in mammalian immune and inflammatory responses, including cytokines, cell adhesion molecules, complement factors, immuno-receptors and anti-apoptotic factors [114]. Progesterone controls endometrial development, in part, by inhibition of the NFκB pathway [115-117]. We propose that the decreased levels of Pgr-A in the eutopic endometrium of baboons with endometriosis mediates the increased levels of NFκB. Increased NFκB may reduce the apoptotic potential of endometriotic tissues. Indeed, eutopic endometrium from women with endometriosis appears to be resistant to apoptosis and shows aberrant expression of genes involved in the apoptotic pathway [118-122]. Furthermore, inhibition of NFκB decreased endometriotic lesion development in the nude mouse model of endometriosis [123]. Inactivation of the NFκB response element in the promoter of the inflammatory cytokine RANTES, which is up-regulated in endometriotic stromal cells, by mutagenesis or progestin treatment leads to suppression of RANTES production in endometriotic and normal endometrial stromal cells [124,125]. Therefore, we propose that increased levels of endometrial NFκB in baboons with endometriosis is mediated by reduced Pgr-A levels and leads to an inflammatory and proliferative type endometrium.

### The AP-1 Family of Transcription Factors in Endometriosis

Micro-array analysis demonstrated a 2.3-fold increase in *c-fos *mRNA in the eutopic endometrium six months following induction of endometriosis [71]. *C-fos *is an estrogen-induced early response gene that mediates estrogen-regulated proliferation in normal and malignant breast and endometrial cells [126-132]. An increased level of *c-fos *in endometriotic endometrium during the window of receptivity provides further evidence to support the hypothesis that the endometrial environment of baboons and women with endometriosis is hyper-estrogenic. Therefore, we examined the expression and distribution of *c-fos *throughout progression of experimental endometriosis in the baboon. C-fos protein was immunolocalized to both the epithelial and stromal cells in normal secretory baboon endometrium. Corresponding to the microarray analysis, increased levels of c-fos protein were present in the eutopic endometrium of animals six months following induction of disease compared to control tissues. Although c-fos protein was present in endometrium from baboons 15 months after inoculation, the level of immunostaining was similar to that seen in control, disease-free endometrium [71]. Likewise, *c-fos *mRNA was differentially expressed in the eutopic endometrium throughout progression of endometriosis. Maximal *c-fos *mRNA expression was observed at three months of disease, with a statistically significant 483-fold up-regulation over control samples. *C-fos *mRNA then gradually decreased throughout progression of disease to a 93- and 60-fold increase at six and 12 months, respectively. Fifteen months after induction of endometriosis levels of *c-fos *mRNA expression were almost the same as those found in control tissues from animals with no disease [71].

C-fos, together with junD and JNK2, are members of the AP-1 family of proteins which play a critical role in controlling cell life and death. In addition to being a transcriptional activator, AP-1 appears to induce gene repression or silencing. Furthermore, c-fos has been shown to inhibit Pgr-induced transcription [133]. As described above, Pgr-A was decreased in the eutopic endometrial epithelial cells within six months of disease [89]. We propose that in the very early stages of disease, when the levels of Pgr-A are similar in endometriotic and control endometrium, that increased levels of *c-fos *may inhibit the ability of Pgr-A to mediate the transcription of progesterone-regulated genes. This inactivity would provide another mechanism whereby Pgr-A activity is inhibited; ESR-1-induced genes, such as MMPs, VEGF, and CYR61, would then be expressed in the endometrium during the window of receptivity, even when there appears to be no differences in the expression level of the SHRs.

*C-fos *transformation of rat fibroblasts induces the expression of DNA 5-methylcytosine transferase (*dnmt1*), which causes hypermethylation and subsequent repression of gene expression by promoting the condensation of chromatin [134-136]. Wu *et al *[137] recently demonstrated that the reduction of HOXA10 mRNA and protein seen in women with endometriosis was associated with hypermethylation of the HOXA10 gene. We have examined the expression of HOXA10 in eutopic endometrium of baboons with experimental endometriosis. We observed a progressive, statistically significant loss of HOXA10 mRNA and protein throughout the progression of endometriosis from three to 15 months [138]. This confirmed preliminary microarray analysis using the Affymetrix gene chip HUM199A, which identified a 2.4-fold down-regulation in HOXA10 mRNA one month after induction of disease [138]. We propose that up-regulation and activity of c-fos seen in the baboon model of endometriosis may induce the expression of *dnmt1 *and subsequent hypermethylation and down-regulation of endometrial HOXA10 and other progesterone-regulated genes.

## Conclusion

**Figure 4:**
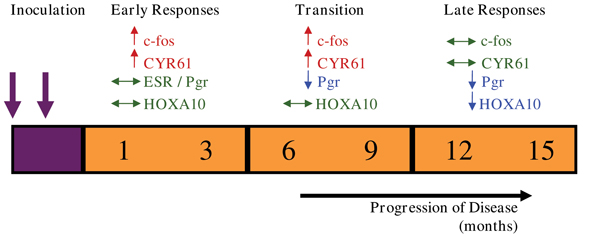
Coordinated Endometrial Changes Associated with Endometriosis. Eutopic endometrial changes induced by the presence of ectopic endometriotic lesions can be classified as early responses, with increased levels of c-fos and CYR61, a transitional period, when the Pgr becomes decreased, and late responses when maintained down-regulation of Pgr may mediate down-regulation of the progesterone-regulated gene, HOXA10. (↑, represents up-regulation, ↓, represents down-regulation, and ↔, represents no change in gene expression.)

We propose that the development of endometriotic lesions within the peritoneal cavity is a highly invasive process. Using the baboon model of experimental endometriosis we have demonstrated that the invasive phenotype is mediated by MMPs. The development of a supporting vasculature is mediated, in part by VEGF and CYR61. The impact of these peritoneal lesions on the eutopic endometrium is clearly illustrated by the reduced level of fertility observed in women and baboons with endometriosis. However, the mechanisms by which the presence of disease alters the eutopic endometrium is unclear. We have demonstrated aberrant expression profiles of many estrogen and progesterone regulated genes during the window of receptivity in baboons with experimental endometriosis. VEGF, CYR61 and c-fos show transient increases during the early stages of disease, while changes in other genes, including ESR1, Pgr-A, and HOXA10, develop at later stages of disease (Figure 4). Furthermore, we have shown irregular responses within the endometrium to embryonic signals. In conclusion, these data suggest that, in an induced model of endometriosis in the baboon, an increased angiogenic capacity, decreased apoptotic potential, progesterone resistance, estrogen hyper-responsiveness, and an inability to respond appropriately to embryonic signals mediates the reduced fecundity associated with this disease.

However, several questions remain to fully understand the impact of endometriotic lesions on the eutopic endometrium. Firstly, are these aberrant gene expression patterns a cause or effect of endometriosis; i.e. does the eutopic endometrium have an inherent abnormal phenotype or is it induced upon translocation to the peritoneal cavity? It is not possible to address this question in women as most present to the clinic with well established disease. We propose that it is the establishment of disease itself that actuates the anomalous activity of the eutopic endometrium. Although the studies in the baboon model of endometriosis described above allow the investigation of the very early stages of disease, current ongoing studies in our laboratory are analyzing the endometrium harvested from the same animal before and after induction of endometriosis to more completely address this question. Secondly, the mechanisms whereby the ectopic endometrium communicates with the eutopic endometrium are unclear. It has been suggested that communication and transfer between the peritoneal and uterine cavities occurs in both directions, such that factors secreted in the peritoneal fluid, which baths the reproduction organs, enters the fallopian tubes and reaches the endometrium. It is also possible that ectopic lesions, which we and others have shown are highly vascularized, may communicate with the eutopic endometrium in an endocrine manner, via their newly formed vascular networks. Thirdly, we have described altered expression patterns of several estrogen and progesterone regulated genes in the early stages of disease progression, when the steroid hormone receptors themselves appear to be normal. Rapid non-genomic effects of estrogen and progesterone have been identified in many mammalian tissues [139-141]. It is possible that transcription of these hormonally regulated genes occurs at a non-genomic level; alternatively, these genes could be regulated by a non-hormonal mechanism.

We believe that the baboon model of experimental endometriosis provides a powerful model to understand the early events associated with the pathophysiology of endometriosis and its associated infertility.

## Competing interests

Neither JMH nor ATF have any competing interests in the data presented in this review.

## Authors' contributions

JMH and ATF contributed equally to this manuscript.

## References

[B1] Eskenazi B, Warner ML (1997). Epidemiology of endometriosis. Obstet Gynecol Clin North Am.

[B2] Gruppo Italiano (1994). Prevalence and anatomical distribution of endometriosis in women with selected gynecological conditions: results from a multicentric Italian Study. Gruppo italiano per lo studio dell'endometriosi. Hum Reprod.

[B3] Sampson JA (1927). Peritoneal endometriosis is due to menstrual dissemination of endometrial tissue into the peritoneal cavity. Am J Obstet Gynecol.

[B4] Blumenkrantz MJ, Gallagher N, Bashore RA, Tenckhoff H (1981). Retrograde menstruation in women undergoing chronic peritoneal dialysis. Obstet Gynecol.

[B5] The survey was carried out by the UK Endometriosis All Party Parliamentary Group and involved 7025 women from 52 countries, who answered a questionnaire either on paper or via the internet between July 2004 and June 2005. The survey is ongoing and data continues to be reviewed for regular updating.

[B6] Sinaii N, Cleary SD, Ballweg ML, Nieman LK, Stratton P (2002). High rates of autoimmune and endocrine disorders, fibromyalgia, chronic fatigue and atopic disease among women with endometriosis: a survey analysis. Hum Reprod.

[B7] Zamah NM, Dodson MG, Stephens LC, Buttram VC, Besch PK, Kaufman RH (1984). Transplantation of normal and ectopic endometrial tissue into athymic nude mice. Am J Obstet Gynecol.

[B8] Bruner KL, Matrisian ML, Ridgers WH, Gortein F, Osteen KG (1997). Suppression of matrix metalloproteinases inhibits establishment of ectopic lesions by human endometrium in nude mice. J Clin Invest.

[B9] Awwad JT, Sayegh RA, Tao XJ, Hassan T, Awwas ST, Isaacson K (1999). The SCID mouse: an experimental model for endometriosis. Hum Reprod.

[B10] Rossi G, Somigliana E, Moschetta M, Santorsola R, Cozzolino S, Filardo P, Salmaso A, Zingrillo B (2000). Dynamic aspects of endometriosis in a mouse model through analysis of implantation and progression. Arch Gynecol Obstet.

[B11] McClure HM, Ridley JM, Graham CE (1971). Disseminated endometriosis in a rhesus monkey (*Macaca mulatta*). J med Assoc Ga.

[B12] Fanton JW, Hubbard GB (1983). Spontaneous endometriosis in a Cynomolgus monkey. Lab Anim Sci.

[B13] Digiacomo RF, Hooks JJ, Sulima MP, Gibbs CJ, Gajdusek DC (1977). Pelvic endometriosis and Simian Foamy Virus Infection in a pigtailed macaque. J Am Vet Med Assoc.

[B14] Merrill JA (1968). Spontaneous endometriosis in the Kenya baboon (*Papio doguera*). Am J Obstet Gynecol.

[B15] Te Linde RW, Scott RB (1950). Experimental endometriosis. Am J Obstet Gynecol.

[B16] Allen E, Peterson LF, Campbell ZB (1954). Clinical and experimental endometriosis. Am J Obstet Gynecol.

[B17] D'Hooghe TM, Bambra CS, Suleman MA, Dunselman GA, Evers HL, Koninckx PR (1994). Development of a model of retrograde menstruation in baboons (*Papio anubis*). Fertil Steril.

[B18] Rier SE, Martin DC, Bowman RE, Dmowski WP, Becker JL (1993). Endometriosis in rhesus monkeys (*Macaca mulatta*) following chronic exposure to 2,3,7,8-tetrachorodibenzo-*p*-dioxin. Fundam Appl Toxicol.

[B19] D'Hooghe TM, Bambra CS, De Jonge I, Lauweryns JM, Koninckx PR (1996). The prevalence of spontaneous endometriosis in the baboon (*Papio anubis*, (*Papio cynocephalus*) increases with the duration of captivity. Acta Obstet Gynecol Scand.

[B20] D'Hooghe TM, Bambra CS, Raeymaekers BM, Koninckx PR (1996). Serial laparoscopies over 30 months show that endometriosis in captive baboons (*Papio anubis*, *Papio cynocephalus*) is a progressive disease. Fertil Steril.

[B21] D'Hooghe TM, Bambra CS, Raeymaekers SCM, De Jonge I, Lauweryns JM, Koninckx PR (1995). Intrapelvic injection of menstrual endometrium causes endometriosis in baboons (*Papio cynocephalus *and *Papio anubis*). Am J Obstet Gynecol.

[B22] D'Hooghe TM, Riday AM, Bambra CS, Suleman MA, Raeymaekers MS, Koninckx PR (1996). The cycle pregnancy rate is normal in baboons with stage I endometriosis but decreased in primates with stage II and III-IV disease. Fertil Steril.

[B23] Fazleabas AT, Donnelly KM, Srinivasan S, Fortman JD, Miller JB (1999). Modulation of the baboon (*Papio anubis*) uterine endometrium by chorionic gonadotrophin during the period of uterine receptivity. Proc Natl Acad Sci USA.

[B24] Fazleabas AT, Soares MJ, Hunt JS (2006). A Baboon Model for Simulating Pregnancy. Methods in Molecular Medicine: Placenta and Trophoblast: Methods and Protocols.

[B25] Koks CAM, Groothuis PG, Dunselman GAJ, de Goeij AFPM, Evers JLH (1999). Adhesion of shed menstrual tissue in an in-vitro model using amnion and peritoneum: a light and electron microscopic study. Hum Reprod.

[B26] Debrock S, Perre SV, Meuleman C, Moerman P, Hill JA, D'Hooghe TM (2002). 2002 In-vitro adhesion of endometrium to autologous peritoneal membranes: effect of the cycle phase and the stage of endometriosis. Hum Reprod.

[B27] Witz CA, Montoya-Rodriguez IA, Schenken RS (1998). Whole explants of peritoneum and endometrium: a novel model of the early endometriosis lesion. Fertil Steril.

[B28] Witz CA, Dechaud H, Montoya-Rodriguez IA, Thomas MR, Nair AS, Centonze VE, Schenken RS (2002). An *in vitro *model to study the pathogenesis of the early endometriosis lesion. Ann NY Acad Sci.

[B29] Witz CA, Cho S, Centonze VE, Montoya-Rodriguez IA, Schenken RS (2003). Time series analysis of transmesothelial invasion by endometrial stromal and epithelial cells using three-dimensional confocal microscopy. Fertil Steril.

[B30] Fazleabas AT, Brudney A, Gurates B, Chai D, Bulun SA (2002). A modified model for endometriosis. Ann N Y Acad Sci.

[B31] Bruner-Tran KL, Eisenberg E, Yeaman GR, Anderson TA, McBean J, Osteen KG (2002). Steroid and cytokine regulation of matrix metalloproteinase expression in endometriosis and the establishment of experimental endometriosis in nude mice. J Clin Endocrinol Metab.

[B32] Wenzl RJ, Heinzl H (1998). Localization of matrix metalloproteinase-2 in uterine endometrium and ectopic implants. Gynecol Obstet Invest.

[B33] Gottschalk C, Malberg K, Arndt M, Schmitt J, Roessner A, Schultze D, Kleinstein J, Ansorge S (2000). Matrix metalloproteinases and TACE play a role in the pathogenesis of endometriosis. Adv Exp Med Biol.

[B34] Chung HW, Wen Y, Chun SH, Nezhat C, Woo BH, Lake Pola M (2001). Matrix metalloproteinase-9 and tissue inhibitor of metalloproteinase-3 mRNA expression in ectopic and eutopic endometrium in women with endometriosis: a rationale for endometriotic invasiveness. Fertil Steril.

[B35] Bruner-Tran KL, Webster-Clair D, Osteen KG (2002). Experimental endometriosis: the nude mouse as a xenographic host. Ann N Y Acad Sci.

[B36] Collette T, Bellehumeur C, Kats R, Maheux R, Mailloux J, Villeneuve M, Akoum A (2004). Evidence for an increased release of proteolytic activity by the eutopic endometrial tissue in women with endometriosis and for involvement of matrix metalloproteinase-9. Hum Reprod.

[B37] Nap AW, Dunselman GAJ, de Goeij AFPM, Evers JLH, Groothuis PG (2004). Inhibiting MMP activity prevents the development of endometriosis in the chicken chorio-allantoic membrane model. Hum Reprod.

[B38] Bruner KL, Matrisian LM, Rodgers WH, Gorstein F, Osteen KG (1997). Suppression of matrix metalloproteinases inhibits establishment of ectopic lesions by human endometrium in nude mice. J Clin Invest.

[B39] Sillem M, Prifti S, Koch A, Neher M, Jauckus J, Runnebaum B (2001). Regulation of matrix metalloproteinases and their inhibitors in uterine endometrial cells of patients with and without endometriosis. Eur J Obstet Gynecol Reprod Biol.

[B40] D'Antonio M, Martelli F, Peano S, Papoian R, Borrelli F (2000). Ability of recombinant human TNF binding protein-1 (r-h-TBP-1) to inhibit the development of experimentally-induced endometriosis in rats. J Reprod Immunol.

[B41] D'Hooghe TM, Nugent NP, Cuneo S, Chai DC, Deer F, Debrock S, Kyama CM, Mihalyi A, Mwenda J (2006). Recombinant human TNFRSF1a (r-hTBP1) inhibits the development of endometriosis in baboons: a prospective, randomized, placebo- and drug-controlled study. Biol Reprod.

[B42] Barrier BF, Bates GW, Leland M, Leach DA, Robinson RD, Propst AM (2004). Efficacy of anti-tumor necrosis factor therapy in the treatment of spontaneous endometriosis in baboons. Fertil Steril.

[B43] McLaren J, Prentice A, Charnock-Jones DS, Smith SK (1996). Vascular endothelial growth factor (VEGF) concentrations are elevated in peritoneal fluid of women with endometriosis. Hum Reprod.

[B44] Mahnke JL, Dawood MY, Huang JC (2000). Vascular endothelial growth factor and interleukin-6 in peritoneal fluid of women with endometriosis. Fertil Steril.

[B45] Kupker W, Schultze-Mosgau A, Diedrich K (1998). Paracrine changes in the peritoneal environment of women with endometriosis. Hum Reprod Update.

[B46] Pizzo A, Salmeri FM, Ardita FV, Sofo V, Tripepi M, Marsico S (2002). Behavior of cytokine levels in serum and peritoneal fluid of women with endometriosis. Gynecol Obstet Invest.

[B47] Barcz E, Rozewska ES, Kaminski P, Demkow U, Bobrowska K, Marianowski L (2002). Angiogenic activity and IL-8 concentrations in peritoneal fluid and sera in endometriosis. Int J Gynaecol Obstet.

[B48] Tabibzadeh S, Becker JL, Parsons AK (2003). Endometriosis is associated with alterations in the relative abundance of proteins and IL-10 in the peritoneal fluid. Front Biosci.

[B49] Punnonen J, Teisala K, Ranta H, Bennett B, Punnonen R (1996). Increased levels of interleukin-6 and interleukin-10 in the peritoneal fluid of patients with endometriosis. Am J Obstet Gynecol.

[B50] Arici A, Matalliotakis I, Goumenou A, Koumantakis G, Vassiliadis S, Mahutte NG (2003). Altered expression of interleukin-18 in the peritoneal fluid of women with endometriosis. Fertil Steril.

[B51] Donnez J, Smoes P, Gillerot S, Casanas-Roux F, Nisolle M (1998). Vascular endothelial growth factor (VEGF) in endometriosis. Hum Reprod.

[B52] Takehara M, Ueda M, Yamashita Y, Terai Y, Hung YC, Ueki M (2004). Vascular endothelial growth factor A and C gene expression in endometriosis. Hum Pathol.

[B53] Goteri G, Lucarini G, Filosa A, Pierantoni A, Montik N, Biagini G, Fabris G, Ciavattini A (2004). Immunohistochemical analysis of vascular endothelial growth factor cellular expression in ovarian endometriomata. Fertil Steril.

[B54] Tan XJ, Lang JH, Liu DY, Shen K, Leng JH, Zhu L (2002). Expression of vascular endothelial growth factor and thrombospondin-1 mRNA in patients with endometriosis. Fertil Steril.

[B55] Fujishita A, Hasuo A, Khan KN, Masuzaki H, Nakashima H (1999). Immunohistochemical study of angiogenic factors in endometrium and endometriosis. Gynecol Obstet Invest.

[B56] Gashaw I, Hastings JM, Jackson KS, Winterhager E, Fazleabas AT (2006). Induced endometriosis in the baboon (*Papio anubis*) increases the expression of the proangiogenic factor CYR61 (CCN1) in eutopic and ectopic endometrium. Biol Reprod.

[B57] Absenger Y, Hess-Stump H, Kreft B, Kratzschmar J, Haendler B, Schutze N, Regidor PA, Winterhager E (2004). Cyr61, a deregulated gene in endometriosis. Mol Hum Reprod.

[B58] Lau LF, Nathans D (1987). Expression of a set of growth-related immediate early genes in BALB/c 3T3 cells: coordinate regulation with c-fos or c-myc. Proc Natl Acad Sci USA.

[B59] Schutze N, Lechner A, Groll C, Siggelkow H, Hufner M, Kohrle J, Jakob F (1998). The human analog of murine cystein rich protein 61 is a 1alpha,25-dihydroxyvitamin D3 responsive immediate early gene in human fetal osteoblasts: regulation by cytokines, growth factors, and serum. Endocrinology.

[B60] Tsai MS, Bogart DF, Li P, Mehmi I, Lupu R (2002). Expression and regulation of Cyr61 in human breast cancer cell lines. Oncogene.

[B61] Hull ML, Charnock-Jones DS, Chan CL, Bruner-Tan KL, Osteen KG, Tom BD, Fan TP, Smith SK (2003). Antiangiogenic agents are effective inhibitors of endometriosis. J Clin Endocrinol Metab.

[B62] Laschke MW, Elitzsch A, Vollmar B, Vajkoczy P, Menger MD (2006). Combined inhibition of vascular endothelial growth factor (VEGF), fibroblast growth factor (FGF) and platelet-derived growth factor, but not inhibition of VEGF alone, effectively suppressed angiogenesis and vessel maturation in endometriotic lesions. Hum Reprod.

[B63] Nap AW, Griffioen AW, Dunselman GA, Bouma-Ter Steege JC, Thijssen VL, Evers JL, Groothuis PG (2004). Antiangiogenesis therapy for endometriosis. J Clin Endocrinol Metab.

[B64] Nap AW, Dunselman GA, Griffioen AW, Mayo KH, Evers JL, Groothuis PG (2005). Angiostatic agents prevent the development of endometriosis-like lesions in the chicken chorioallantoic membrane. Fertil Steril.

[B65] Becker CM, Sampson DA, Rupnick MA, Rohan RM, Efstathiou JA, Short SM, Taylor GA, Folkman J, D'Amato RJ (2005). Endostatin inhibits the growth of endometriotic lesions but does not affect fertility. Fertil Steril.

[B66] Becker CM, Sampson DA, Short SM, Javaherian K, Folkman J, D'Amato RJ (2006). Short synthetic endostatin peptides inhibit endothelial migration in vitro and endometriosis in a mouse model. Fertil Steril.

[B67] Dabrosin C, Gyorffy S, Margetts P, Ross C, Gauldie J (2002). Therapeutic effect of angiostatin gene transfer in a murine model of endometriosis. Am J Pathol.

[B68] Hii LL, Rogers PA (1998). Endometrial vascular and glandular expression of integrin alpha(v)beta3 in women with and without endometriosis. Hum Reprod.

[B69] Kim SH, Choi YM, Chae HD, Kim KR, Kim CH, Kang BM (2001). Increased expression of endoglin in the eutopic endometrium of women with endometriosis. Fertil Steril.

[B70] Healy DL, Rogers PA, Hii L, Wingfield M (1998). Angiogenesis: a new theory for endometriosis. Hum Reprod Update.

[B71] Hastings JM, Jackson KS, Mavrogianis PA, Fazleabas AT (2006). The estrogen early response gene FOS is altered in a baboon model of endometriosis.. Biol Reprod.

[B72] Salcedo R, Young HA, Ponce ML, Ward JM, Kleinman HK, Murphy WJ, Oppenheim JJ (2001). Eotaxin (CCL11) induces in vivo angiogenic responses by human CCR3 ^+ ^endothelial cells. J Immunol.

[B73] Jones RL, Hannan NJ, Kaitu'u TJ, Zhang J, Salamonsen LA Identification of chemokines important for leukocyte recruitment to the human endometrium at the times of embryo implantation and menstruation. J Clin Endocrinol Metab.

[B74] Ayers JW, Birenbaum DL, Menon KM (1987). Luteal phase dysfunction in endometriosis: elevated progesterone levels in peripheral and ovarian veins during the follicular phase. Fertil Steril.

[B75] Halme J, Becker S, Haskill S (1987). Altered maturation and function of peritoneal macrophages: possible role in pathogenesis of endometriosis. Am J Obstet Gynecol.

[B76] Mills MS, Eddowes HA, Cahill DJ, Fahy UM, Abuzeid MI, McDermott A, Hull MG (1992). A prospective controlled study of in-vitro fertilization, gamete intra-fallopian transfer and intrauterine insemination combined with superovulation. Hum Reprod.

[B77] Witz CA, Montoya IA, Dey TD, Schenken RS (1994). Characterization of lymphocyte subpopulations and T cell activation in endometriosis. Am J Reprod Immunol.

[B78] Fedele L, Bianchi S, Marchini M, Franchi D, Tozzi L, Dorta M (1996). Ultrastructural aspects of endometrium in infertile women with septate uterus. Fertil Steril.

[B79] Lessey BA, Metzger DA, Haney AF, McCarty KS (1989). Immunhistochemical analysis of estrogen and progesterone receptors in endometriosis: comparison with normal endometrium during the menstrual cycle and the effect if medical therapy. Fertil Steril.

[B80] Prentice A, Randall BJ, Weddell A, McGill A, Henry L, Horne CH, Thomas EJ (1992). Ovarian steroid receptor expression in endometriosis and in two potential parent epithelia: endometrium and peritoneal mesothelium. Hum Reprod.

[B81] Bergqvist A, Ferno M (1993). Oestrogen and progesterone receptors in endometriotic tissue and endometrium: comparison between different cycle phases and age. Hum Reprod.

[B82] Jones RK, Bulmer JN, Searle RF (1995). Immunohistochemical characterization of proliferations, oestrogen receptor and progesterone receptor expression in endometriosis: comparison of eutopic and ectopic endometrium with normal cycling endometrium. Hum Reprod.

[B83] Nisolle M, Casanas-Roux F, Wyns C, De Menten Y, Mathieu PE, Donnez J (1994). Immunohistochemical analysis of estrogen and progesterone receptors in endometrium and peritoneal endometriosis: a new quantitative method. Fertil Steril.

[B84] Lessey BA, Castelbaum AJ, Sawin SW, Buck CA, Schinnar R, Bilker W, Strom BL (1994). Aberrant integrin expression in the endometrium of women with endometriosis. J Clin Endocrinol Metab.

[B85] Lessey BA, Castelbaum AJ (1995). Integrins in the endometrium of women with endometriosis. Br J Obstet Gynaecol.

[B86] Ota H, Tanaka T (1997). Integrin adhesion molecules in the endometrial glandular epithelium in patients with endometriosis and adenomyosis. J Obstet Gynaecol Res.

[B87] Taylor HS, Bagot C, Kardana A, Olive D, Arici A (1999). *HOX *gene expression is altered in the endometrium of women with endometriosis. Hum Reprod.

[B88] Gui Y, Zhang J, Yuan L, Lessey BA (1999). Regulation of HOXA-10 and its expression in normal and abnormal endometrium. Mol Hum Reprod.

[B89] Jackson KS, Hastings J, Brudney A, Fazleabas A (2004). Endometriosis alters the pattern of steroid receptor expression in the baboon endometrium. Biol Reprod.

[B90] Cooke PS, Buchanan DL, Young P, Setiawan T, Brody J, Korach KS, Taylor J, Lubahn DB, Cunha GR (1997). Stromal estrogen receptors mediate mitogenic effects of estradiol on uterine epithelium. Proc Natl Acad Sci USA.

[B91] Giess R, Tanasescu I, Steck T, Sendter M (1999). Leukaemia inhibitory factor gene mutations in infertile women. Mol Hum Reprod.

[B92] Sherwin JR, Smith SK, Wilson A, Sharkey AM (2002). Soluble gp130 is up-regulated in the implantation window and shows altered secretion in patients with primary unexplained infertility. J Clin Endocrinol Metab.

[B93] Karpovich N, Klemmt P, Hwang JH, McVeigh JE, Heath JK, Barlow DH, Mardon HJ (2004). The production of interleukin-11 and decidualization are comprised in endometrial stromal cells derived from patients with infertility. J Clin Endocrinol Metab.

[B94] D'Hooghe TM, Bambra CS, Wiao L, Peixe K, Hill JA (2001). Effect of menstruation and intrapelvic injection of endometrium on inflammatory parameters of peritoneal fluid in the baboon (*Papio anubis *and *Papio *cynocephalus). Am J Obstet Gynecol.

[B95] Gazvani R, Templeton A (2002). Peritoneal environment, cytokines, and angiogenesis in the pathophysiology of endometriosis. Reproduction.

[B96] Harada T, Enatsu A, Mitsunari M, Nagano Y, Ito M, Tsudo T, Taniguchi F, Iwabe T, Tanikawa M, Terakawa N (1999). Role of cytokines in progression of endometriosis. Gynecol Obstet Invest.

[B97] Mazzeo D, Vigano P, Di Blasio AM, Sinigaglia F, Vignali M, Panina-Bordignon P (1998). Interleukin-12 and its free p40 subunit regulate immune recognition of endometrial cells: potential role in endometriosis. J Clin Endocrinol Metab.

[B98] Levia MC, Hasty LA, Pfeifer S, Mastroinni L, Lyttle CR (1993). Increased chemotactic activity of peritoneal fluid in patients with endometriosis. Am J Obstet Gynecol.

[B99] McLaren J, Prentice A, Charnock-Jones DS, Milligan SA, Muller KH, Sharkey AM, Smith SK (1996). Vascular endothelial growth factor is produced by peritoneal fluid macrophages in endometriosis and is regulated by ovarian steroids. J Clin Invest.

[B100] Dmowski WP (1995). Immunological aspects of endometriosis. Int J Gynaecol Obstet.

[B101] Oral E, Arici A (1997). Pathogenesis of endometriosis. Obstet Gynecol Clin North Am.

[B102] Kao LC, Germeyer A, Tulac S, Lobo S, Yang JP, Taylor RN, Osteen K, Lessey BA, Giudice LC (2003). Expression profiling of endometrium from women with endometriosis reveals candidate genes for disease-based implantation failure and infertility. Endocrinology.

[B103] Fazleabas AT, Brudney A, Chai D, Langoi D, Bulun SE (2003). Steroid receptor and aromatase expression in baboon endometriotic lesions. Fertil Steril.

[B104] Hauserman HM, Donnelly KM, Bell SC, Verhage HG, Fazleabas A (1998). Regulation of the glycosylated β-lactoglobulin homolog, glycodelin (placental protein 14(PP14)) in the baboon (*Papio anubis*) uterus. J Clin Endocrinol Metab.

[B105] Waites GT, Bell SC (1989). Immunological localization of human pregnancy-associated endometrial α2-globulin, a glycosylated β-lactoglobulin homologue, in decidua and placenta during pregnancy. J Reprod Fertil.

[B106] Kamarainen M, Leivo I, Julkunen M, Seppala M (1993). Localization of progesterone-associated endometrial protein mRNA by in-site hybridization in human pregnancy decidua, endometriosis and borderline endometroid adenoma. J Mol Endocrinol.

[B107] Waites GT, Bell SC, Walker RA, Wood PL (1990). Immunohistological distribution of the secretory endometrial protein, 'pregnancy-associated endometrial α2-globulin, a glycosylated β-lactoglobulin homologue, in the human fetus and adult employing monoclonal antibodies. Hum Reprod.

[B108] Li TC, Dalton C, Hunjan KS, Warren MA, Bolton AE (1993). The correlation of placental protein 14 concentrations in uterine flushings and endometrial morphology in the peri-implantation period. Hum Reprod.

[B109] Li TC, Ling E, Dalton C, Bolton AE, Cooke ID (1993). Concentration of endometrial protein PP14 in uterine flushings throughout the menstrual cycle in normal, fertile women. Br J Obstet Gynaecol.

[B110] Dalton CF, Laird SM, Serle F, Saravelos H, Warren MA, Li TC, Bolton AE (1995). The measurement of CA125 and placental protein 14 in uterine flushings in women with recurrent miscarriage; relation to endometrial morphology. Hum Reprod.

[B111] Simon C, Valbuena D, Krussel J, Bernal A, Murphy CR, Shaw T, Pellicer A, Polan ML (1998). Interleukin-1 receptor antagonist prevents embryonic implantation by a direct effect on the endometrial epithelium. Fertil Steril.

[B112] Tanaka T, Umesaki N, Mizuno K, Chang L, Ohtaki S, Ogita S (1998). Enhancement of apoptotic susceptibility by interleukin-1 beta in human endometrial epithelial cells. Gynecol Endocrinol.

[B113] Strakova Z, Mavrogianis P, Meng X, Hastings JM, Jackson KS, Cameo P, Brudney A, Knight O, Fazleabas AT (2005). *In vivo *infusion of interleukin-1β and chorionic gonadotropin induces endometrial changes that mimic early pregnancy events in the baboon. Endocrinology.

[B114] McKay LI, Cidlowski JA (1999). Molecular Control of Immune/Inflammatory Responses: Interactions between nuclear factor-κB and steroid receptor-signaling pathways. Endocr Rev.

[B115] Kelly RW, King AE, Critchley HOD (2001). Cytokine control in human endometrium. Reproduction.

[B116] King AE, Critchley HO, Kelly RW (2001). The NF-kappaB pathway in human endometrium and first trimester decidua. Mol Hum Reprod.

[B117] Davies S, Dai D, Feldman I, Pickett G, Leslie KK (2004). Identification of a novel mechanism of NF-kappaB inactivation by progesterone through progesterone receptors in Hec50co poorly differentiated endometrial cancer cells: induction of A20 and ABIN-2. Gynecol Oncol.

[B118] Johnson MC, Torres M, Alves A, Bacallao K, Fuentes A, Vega M (2005). Augmented cell survival in eutopic endometrium from women with endometriosis: expression of c-myc, TGF-beta1 and bax genes. Reprod Biol Endocrinol.

[B119] Braun DP, Ding J, Shen J, Rana N, Fernandez BB, Dmowski WP (2002). Relationship between apoptosis and the number of macrophages in eutopic endometrium from women with and without endometriosis. Fertil Steril.

[B120] Dmowski WP, Ding J, Shen J, Rana N, Fernandez BB, Braun DP (2001). Apoptosis in endometrial glandular and stromal cells in women with and without endometriosis. Hum Reprod.

[B121] Meresman GF, Vighi S, Buquet RA, Contreras-Ortiz O, Tesone M, Rumi LS (2000). Apoptosis and expression of Bcl-2 and Bax in eutopic endometrium from women with endometriosis. Fertil Steril.

[B122] Imai A, Takagi A, Tamaya T (2000). Gonadotropin-releasing hormone analog repairs reduced endometrial cell apoptosis in endometriosis in vitro. Am J Obstet Gynecol.

[B123] Gonzalez RR, Van Langendonckt S, Defrere S, Mettlen M, Donnez J (2005). Effect of NF-κB inhibition on the development of endometriosis in a nude mouse model. Eur J Ob Gyn.

[B124] Zhao D, Lebovic DI, Taylor RN (2002). Long-term progestin treatment inhibits RANTES (regulated on activation, normal T cell expressed and secreted) gene expression in human endometrial stromal cells. J Clin Endocrinol Metab.

[B125] Lebovic DI, Chao VA, Martini JF, Taylor RN (2001). IL-1beta induction of RANTES (regulated upon activation, normal T cell expressed and secreted) chemokine gene expression in endometriotic stromal cells depends on a nuclear factor-kappaB site in the proximal promoter. J Clin Endocrinol Metab.

[B126] Reis FM, Ribeiro MFM, Spritzer PM (1999). Regional localization of immunoreactive *c-fos *and prolactin in human endometrium during the normal menstrual cycle. Gynecol Obstet Invest.

[B127] Mendoza-Rodriguez CA, Merchant-Larios H, Segura-Valdez ML, Moreno-Mendoza N, Cruz ME, Arteaga-Lopez P, Camacho-Arroyo I, Dominguez R, Cerbon M (2003). *c-fos *and estrogen receptor gene expression pattern in the rat uterine epithelium during the estrous cycle. Mol Reprod Dev.

[B128] Cicatiello L, Ambrosini C, Coletta B, Scalona M, Sica V, Bresciani F, Weisz A (1992). Transcriptional activation of jun and actin genes by estrogen during mitogenic stimulation of rat uterine cells. J Steroid Biochem Mol Biol.

[B129] Cicatiello L, Sica V, Bresciani F, Weisz A (1993). Identification of a specific pattern of "immediate-early" gene activation induced by estrogen during mitogenic stimulation of rat uterine cells. Receptor.

[B130] Duan R, Xie W, Burghardt RC, Safe S (2001). Estrogen receptor-mediated activation of the serum response element in MCF-7 cells through MAPK-dependent phosphorylation of Elk-1. J Biol Chem.

[B131] Duan R, Xie W, Li A, McDougal A, Safe S (2002). Estrogen regulation of *c-fos *gene expression through phosphatidyl-3-kinase-dependent activation of serum response factor in MCF-7 breast cancer cells. Biochem Biophys Res Comm.

[B132] Nemos C, Delage-Mourroux R, Jouvenot M, Adami P (2004). Onset of direct 17-β estradiol on proliferation and *c-fos *expression during oncogenesis of endometrial glandular epithelial cells. Exp Cell Res.

[B133] Shemshedini L, Knauthe R, Sassone-Corsi P, Pornon A, Gronemeyer H (1991). Cell specific inhibitory and stimulatory effects of fos and jun proteins on transcription activation of nuclear factors. EMBO J.

[B134] Bakin AV, Curran T (1999). Role of DNA 5-Methylcytosine transferase in cell transformation by *fos*. Science.

[B135] Nan X, Ng HH, Johnson CA, Laherty CD, Turner BM, Eisenman RN, Bird A (1998). Transcriptional repression by the methyl-CpG-binding protein MeCP2 involves a histone deacetylase complex. Nature.

[B136] Jones PL, Veenstra GJ, Wade PA, Vermaak D, Kass SU, Landsberger N, Strouboulis J, Wolffe AP (1998). Methylated DNA and MeCP2 recruit histone deacetylase to repress transcription. Nat Genet.

[B137] Wu Y, Halverson G, Basir Z, Strawn E, Yan P, Guo S-W (2005). Aberrant methylation at HOXA10 may be responsible for its aberrant expression in the endometrium of patients with endometriosis. Am J Obstet Gynecol.

[B138] Fazleabas A, Sarno J, Jackson K, Hamilton A, Talbi S, Giudice L, Taylor H (2005). Endometrial HOXA10 expression is decreased in baboons with endometriosis. J Soc Gynecol Investig.

[B139] Luconi M, Francavilla F, Porazzi I, Macerola B, Forti G, Baldi E (2004). Human spermatozoa as a model for studying membrane receptors mediating rapid nongenomic effects of progesterone and estrogen. Steroids.

[B140] Simoncini T, Mannella P, Fornari L, Caruso A, Varone G, Genazzani AR (2004). Genomic and non-genomic effects of estrogens on endothelial cells. Steroids.

[B141] Vasudevan N, Kow LM, Pfaff D (2005). Integration of steroid hormone initiated membrane action to genomic function in the brain. Steroids.

